# Foreign Summary

**Published:** 1839-03-02

**Authors:** 


### FOREIGN SUMMARY.

*Musk and Gum Ammoniac in Tympanitis.*—Dr. Tradini recommends highly a combination of moschus et gum. ammoniac. in tympanitis in the following proportion:

Mosch. gr. iij.
Gum. ammon. gr. xii.

M. Fiant pilulæ tres. D. P. Take one pill morning, noon, and evening.

The remedy is most proper where there is much weakness and debility, which generally accompany the above disease. (Il filiatre sebazio.)

Moxæ and caustics applied on the head in hydrocephalus acutus in children.

Against the above disease, Dr. Constant, in Paris, (*Bullet. de Therapeutique, Med. et. Chir.*,) recommends, particularly, moxæ applied on the head, on each side of the sutura sagittalis, and assures us, that he has by this treatment been able to save several children. Dr. Dürr, in Hall, in Wurtemburg, who has lately written on this disease, has derived great benefit from the use of caustics in this disease. (*Frorieps Notiz.*, No. 1021, Feb., 1836.) He shaves the head, where the sutura sagittalis and lambdoidea come together, and puts on a small emplastrum, about as large as an American dollar, (silver dollar,) spread thinly with the following unguent:

Ungu. acris Autenriethi ʒj.

Tartar. emetic.

Ungu. canthar. āā ʒss.

After four or six hours the skin is raised without there having been any considerable pain. The plaster is spread again, and when, after six or twelve hours, he observes water, he makes an incision, lets the water run out, and applies, every twelve hours, an ointment made up from

Ungu. basilic.
Emplast. de minio āā.

After twenty-four hours, you will have produced an artificial, good looking ulcer. In cases where the suppuration is not great, or seems to cease, Dr. Dürr applies a combination of both the above ointments.—*Bibliothec. for Læger, Copenhagen.*

---

*Remarks on, with Case of, Pneumo-thorax.*—The following case lately occurred in the practice of M. Chomel, at the Hôtel Dieu.

A man, affected with pulmonary tubercles in a state of softening, was suddenly seized with a violent pleuritic pain or stitch, which caused extreme difficulty in breathing, and considerable febrile action in the whole system.

On examining the chest, it was observed to be altogether fuller and more capacious than it had been, and at the same time, to be much more resonant on percussion, while the respiratory murmur had become much more indistinct.

When lecturing upon this case, M. Chomel took occasion to state that, in his opinion, the disease of *pneumo-thorax* is never idiopathic,—or, in other words, that air is never secreted from the pleural surfaces—but is always the result of a communication between the air cells of the lungs and the bag of the pleura. Such a communication may take place in one of two ways; either by the ulceration of a vomica outwards, as in the present case, or in consequence of a purulent effusion in the pleural cavity being followed by an ulceration at some point of the lungs.

He stated, at the same time, that he took the same view of tympanites abdominalis—the effusion of air being, according to him, always the result of an intestinal perforation.

Such a perforation may have taken place either from within outwards, as is occasionally the case in some cases of typhus fever, or from without inwards, as now and then happens in consequence of a purulent collection in the abdominal cavity. An instance of this sort occurred very recently in M. Chomel's clinique, in a woman, who died from an immense abscess in the pelvis: ulceration had taken place at one point of the large intestine, and had penetrated through all its coats, except the mucous one.

It is necessary to distinguish the peritoneal tympanites, which we not unfrequently meet with in dissection, when there is certainly no perforation of the intestines, from that *alleged* or *presumed form* of the disease, which has been attributed to the secretion of air during life: it is entirely owing to the incipient cadaveric decomposition. M. Chomel mentioned a very remarkable instance of this cadaveric tympanites, which he recently met with. A restaurateur in Paris, who was immensely fat, but seemed to be in very good health, was most unexpectedly found dead in his bed. The body was examined thirty hours after death. The season was summer. No sooner were the abdominal parietes divided, than a loud explosion, which M. Chomel compares to that produced by the discharge of an air-gun, was heard; so violent was the rush of the confined air from the aperture which had been made into the cavity of the abdomen.

But now to return to our case of pneumo-thorax.

On the following day, after the presumed rupture of the vomica, and the communication between the air cells of the lungs and the pleural cavity had taken place, the expansion and also the resonance of the thoracic parietes were found to have considerably increased.

In proportion, as the pneumo-thorax was more decided, the auscultatory signs became more and more distinct and decisive. At the lower part of the chest, a sound, analogous to the amphoric bruit, was perceptible; and more externally, a distinct metallic tinckling was to be heard. Over the scapular region, a *bruit de secousse*, such as is caused by striking a drum with the finger, was audible when the patient spoke.

Along with these symptoms, there was extreme anxiety and difficulty of breathing, amounting to orthopnœa, &c., and, as we have mentioned above, the chest was remarkably resonant on percussion.

The treatment which M. Chomel adopts in almost all cases of internal perforation, whether of the thoracic or of the abdominal viscera, consists in the exhibition of opiates, until they produce a decided narcotism of the system. The object is not only to quiet the pain which is almost always present, but also to bring on a state of *inertia* of the whole system, so as to permit nature to exercise her own medicative and reparatory efforts.

From the results of several cases of presumed intestinal perforation, this treatment certainly seems to be by far the most advisable.—*Med. Chirur. Rev. for January, from Lançette Française.*

---

*Poisoning by Arsenic—Exhumation of the Body, and Conviction of the Criminal, three years after death.*—The following case may be added to those reported by M. Orfila and others, where the assistance of chemistry has most strikingly led to the detection of crime, many months and even years after its perpetration.

A widow woman, by name Lamothe, had, in November, 1833, mentioned to several of her acquaintances that a Madame Chevalier had made her universal legatee, and that she hoped she might be soon in possession of her property, as her friend "ne pouvait pas vivre longtemps." It was not long after the date of this foolish speech that Madame Chevalier did in truth die, after a severe attack of cholic, accompanied with most distressing vomiting.

Public rumour, at the time, accused Lamothe of having committed murder; but it does not seem that it ever reached the ear of the public authorities.

Three years afterwards, the house adjoining to that of Lamothe took fire; and the inhabitants of the village, having never forgotten the tale of Madame Chevalier's death, did not hesitate to accuse her (Lamothe) now of wilful arson.

The magistrates, hearing of these reports, determined to investigate the matter minutely. It was ascertained that Lamothe had some arsenic in her possession at the time of Madame Chevalier's death. They ordered the body to be immediately disinterred, three years exactly having intervened since the period of her death.

The soil of the burying-ground being remarkably dry, the body was found not so much decomposed as might have been expected. It was forthwith sent off to Paris, to be examined, pathologically and chemically, by Drs. Barruel, Henry and Olivier, three distinguished men of science in the metropolis. On examining the trunk, it was found completely dry, mummyfied, and almost inodorous; the abdominal parietes, although much shrivelled, were entire. On removing these, there seemed to be no vestiges of the viscera, in consequence of the extreme desiccation which had taken place. On examining, however, the parts more attentively, the intestines were found to be reduced to the state of mere membranous laminæ, adhering to each other, and to the spine and edges of the pelvis. In the interstices of these membranous *folia*, a brownish coloured pulverulent matter was observed. All the viscera, parenchymatous, such as the liver and spleen, as well as the fleshy and membranous as the uterus and intestines, had become blended together, just as now described.

At one spot only, on the upper two lumbar vertebræ, there was a small portion of a brown substance, waxy in consistence, which was probably a portion of the liver. After these short details, it is unnecessary to add that it was not possible to distinguish the abdominal viscera, one from another.

A small fragment of the diaphragm, on the right side, still remained. The only vestige of the lungs was a brown friable substance, in irregular nodules, and the heart was converted into a hard, black, and fragile mass, resting on the vertebræ.

The entire contents of the abdominal cavity—if such it can be called—were now carefully scraped from the bones of the spine and pelvis, and collected together, the earthly granular matter being separated from the foliaceous or membranous substance.

The earthy matter was first examined. A portion was boiled in distilled water, acidulated with a small quantity of pure hydrochloric acid.

On cooling, the liquor was filtered. A small quantity of brown deposit was collected on the blotting-paper, and the filtered liquor was of the same colour.

Through a portion of the latter, diluted with water, a stream of pure sulphuretted hydrogen gas was caused to pass for the space of two hours. This gave rise to a light brownish precipitate, which was carefully collected and tested. It gave no traces of any metallic sulphuret, and was found to contain nothing but minute portions of chloruret of sodium, phosphate of lime, and oxide of iron—the usual salts of animal substances.

The deposit on the filtering paper was then dried and calcined in a crucible; the residue was then examined, and found to contain only the salts now mentioned, and to give no signs of metallic sulphuret. Hitherto, therefore, nothing had been detected to lead to any suspicion of poisoning.

The shrivelled viscera were now subjected to a most careful examination. They were first macerated in rectified alcohol for forty-eight hours, and then withdrawn. The spirit, which had acquired a greenish brown colour, was filtered and distilled in a glass retort. The residue was concentrated in a water-bath to the consistence of a soft extract, and then treated with slightly acidulated water, and boiled. Ammonia was added to neutralize the acid; and then a stream of sulphuretted hydrogen was passed through the fluid for a length of time. A deep-brown precipitate was formed in consequence. This was carefully collected on filtering paper; and the strained liquor was evaporated to dryness, until a dark brown residue was obtained. On burning a small portion of this on live coals, an empyreumatic animal odour, followed by one resembling garlic, was perceived.

Another portion was then heated strongly with hydrochloric acid, until all the brown matter had disappeared; and the dry product of this treatment. being first neutralized, gave a bright-red precipitate with nitrate of silver, similar to that afforded by the action of this salt on any of the preparations of arsenic.

There were, therefore, strong suspicions that arsenic would be discovered in the precipitated matter collected on the filter.

It is unnecessary to particularise the experiments both in the *dry* and in the *wet* method, which were performed. Suffice it to say, that they proved beyond all doubt the existence of this poison. The commissioners sent to the public authorities, along with their report, specimens of—1, arsenic reduced to a metallic state; 2, arseniate of silver; and 3, sulphuret of arsenic floating in water.

The Court of Assizes at once pronounced judgment against the woman Lamothe, and condemned her to perpetual imprisonment. This took place on the 17th of March, 1837—three years and a half after the death of Madame Chevalier.

In closing this brief report, it only remains to add that the experiments necessary in such a case as the preceding, require the most patient and protracted care and delicacy. A week, at least, is not too long for such an investigation.—*Ib. from Annales d'Hygiene, &c.*

---

*New Theory and Treatment of Erysipelas.* By M. Blandin, Surgeon to the Hôtel Dieu, Paris.—Erysipelas may be divided into two varieties, according to the causes which give rise to it. These may be either external or internal; as a wound or injury, or a disordered state of the constitution generally; and the disease will vary considerably in its characters as the one or the other cause may occasion it. According to M. Blandin's theory, that variety which is excited by external injury is *at first* a local affection, and afterwards tends to diffuse itself generally; the fluids, which are altered by the diseased actions going on in the part, having a concentric course, and thus spread themselves through the whole system and excite violent reaction. Erysipelas, on the contrary, arising from an internal cause, is at first generally diffused, and has a tendency to become localized; nature making an effort to determine the disturbing influence towards a single point.

With regard to the anatomical nature or proximate cause of erysipelas, M. B. considers that it consists in acute inflammation of the minute lymphatic vessels of the skin, which are first affected, and inflammation of the substance of the skin itself afterwards follows. [This idea of the absorbents being inflamed in erysipelas is not new, though M. B. is the first who has extensively applied it, in numerous cases, both to pathology and therapeutics: this same theory has been entertained by several authors; among others, MM. Ribes, Dance, and Chomel.] The degree of inflammation between the lymphatic capillaries and the substance of the skin is not always in equal proportion. In erysipelas from internal causes, it is the latter which predominates; in the traumatic variety, the absorbents are principally affected: and this causes the difference in the seriousness of the affections; for according to M. B., physicians generally find erysipelas a trifling disorder, while surgeons regard it as a most serious disease.

M. Blandin's treatment is founded on the principle that the pre-existing and predominant affection is inflammation of the lymphatics, and that, when this is checked, there only remains simple inflammation of the integuments. As the disease is propagated towards those lymphatic ganglions which are situated most centrically, or nearest to the trunk, it is here that we should commence the treatment, which consists in successive applications of leeches over the absorbent glands, and not on the erysipelatous surface of the skin itself; as in the latter case they only weaken the patient. This plan is principally applicable to the traumatic variety, when situated in the extremities; but he considers that it may be also employed with advantage when the skin of the trunk is affected, and when the disease arises from internal causes.

M. B. has applied his plan of treatment to a great number of cases, and informs us that, during two years that he has employed it, he has scarcely lost a single patient; but he ought to publish the exact number of instances in which he has tried it, with the particulars of the different cases: this would render his statements much more valuable.—*Brit. and For. Med. Rev. for January, from Journal des Connaissances Médico-chirurgicales.* July, 1837.

---

*Recovery from a Wound of the Ascending Aorto.* By Dr. Heil.—Joseph Hoffman, æt. thirty-two, a Bavarian soldier, while engaged in a drunken quarrel in the year 1812, received a stab in the left side. The weapon, which was a common table-knife, penetrated the thorax between the fifth and sixth ribs. The left lung was wounded, and the stab was followed by copious hemorrhage. He remained some time exposed to the cold air, and when the doctor saw him, which was about an hour after the receipt of the wound, he was lying still bleeding but apparently lifeless. He was removed to the hospital, and the edges of the wound brought together by plaster, although blood still continued to ooze. After having remained some hours unconscious he came to himself, but was then amaurotic, probably from the abundant depletion which he had undergone. In about four months the external wound had entirely healed, but the amaurosis in spite of all the remedies employed still continued; and in this state he left the hospital. He led it appears an irregular life, and some months afterwards was again brought into the hospital labouring under an attack of pneumonia, of which he died in the year 1813. On examining his body, the cicatrix of the wound in the chest was plainly visible: in laying open the cavity, it was found that the correspondent part of the lung was adherent to the pleura, and that the cicatrix extended through the whole substance of the lung to the back part of the organ, where the weapon must have passed out. From this point the knife appeared to have extended upwards towards the ascending aorta; and in this vessel there was an opening with an irregular border about a quarter of an inch in length, which was occupied by a firm coagulum of blood, (thrombus.) On laying open the vessel the weapon was found to have penetrated completely into its cavity. There was a rounded cicatrix on the inside completely close d up as on the outside. The extraordinary cure had probably taken place from a coagulum having formed during the syncope, into which the man was plunged after receiving the wound, aided by the cold to which he was exposed.

[The description of the cicatrix in the aorta is not very clearly given; but the case is altogether very remarkable, as it shows that wounds of this important vessel are not always *absolutely* mortal, as they are laid down in some systems of classification.]—*Ib. from Henke's Zeitschrift.* 1837–8.

---

*On the immoveable Fracture-bandage.* Dy Dr. Fricke.—Dr. Fricke has given reports of sixteen cases of fracture successfully treated by means of Seutin's starch-bandage. He applies it by first rolling the limb with a common broad bandage, over which a layer of starch is placed. On this two pasteboard splints, the length and shape of the fractured limb, and nearly encirling it, are applied wet; then a layer of starch, and over this a roller, after each turn of which the starch is applied; the whole being covered with paper, to prevent its sticking. Fricke has usually found the application of this bandage successful.

The following are some of the advantages attending the use of this bandage:—1. The materials are readily procurable. 2. The apparatus lies close to the inequalities of the extremity. 3. Any part of it can be renewed without disturbing the other parts. 4. It is light, and does not prevent the use of the extremity. 5. The limb is so firmly supported, that in two days the patient can leave his bed and walk about.

If the lower limb be fractured, it should be supported by means of a sling round the neck. In applying this bandage, Fricke recommends the surgeon to wait for the subsidence of swelling and inflammation. He does not think, with Seutin and Velpeau, that the patient might even undertake a journey immediately after the injury when the bandage is dry.—*Ib. from Hamburg Zeitschrift f. d. g. Med. April,* 1838.

